# A review of bioenergetic modelling for marine mammal populations

**DOI:** 10.1093/conphys/coac036

**Published:** 2022-06-21

**Authors:** Enrico Pirotta

**Affiliations:** Centre for Research into Ecological and Environmental Modelling, University of St Andrews, St Andrews KY16 9LZ, UK

**Keywords:** population consequences of disturbance, pinnipeds, individual-based modelling, energy budgets, cetaceans, bioenergetic models

## Abstract

Bioenergetic models describe the processes through which animals acquire energy from resources in the environment and allocate it to different life history functions. They capture some of the fundamental mechanisms regulating individuals, populations and ecosystems and have thus been used in a wide variety of theoretical and applied contexts. Here, I review the development of bioenergetic models for marine mammals and their application to management and conservation. For these long-lived, wide-ranging species, bioenergetic approaches were initially used to assess the energy requirements and prey consumption of individuals and populations. Increasingly, models are developed to describe the dynamics of energy intake and allocation and predict how resulting body reserves, vital rates and population dynamics might change as external conditions vary. The building blocks required to develop such models include estimates of intake rate, maintenance costs, growth patterns, energy storage and the dynamics of gestation and lactation, as well as rules for prioritizing allocation. I describe how these components have been parameterized for marine mammals and highlight critical research gaps. Large variation exists among available analytical approaches, reflecting the large range of life histories, management needs and data availability across studies. Flexibility in modelling strategy has supported tailored applications to specific case studies but has resulted in limited generality. Despite the many empirical and theoretical uncertainties that remain, bioenergetic models can be used to predict individual and population responses to environmental change and other anthropogenic impacts, thus providing powerful tools to inform effective management and conservation.

## Teaser

Bioenergetic models describe energy acquisition and allocation. In marine mammals, they have been used to quantify prey consumption and predict the consequences of disturbance. I describe how model components have been parameterized and highlight research gaps. Despite a lack of generality, bioenergetic modelling provides a powerful management and conservation tool.

## Introduction

The rates at which animals extract energy from the environment and use it to fuel different life functions affect their ability to survive, grow and reproduce. Energy thus acts as an effective proximate currency to integrate diverse biological and ecological processes influencing an individual’s fitness (Sibly *et al.,* 2013). In turn, modelling the mechanisms that underpin individual energy management can help quantify broader processes with cascading effects on ecological communities, such as total resource consumption by populations (e.g. Delmas *et al.,* 2017), competition between species (e.g. Caut *et al.,* 2006), changing environmental conditions (e.g. Humphries *et al.,* 2004) and trait-mediated indirect interactions between predators and prey (Werner and Peacor, 2003). Moreover, disturbance from human activities might affect the rate of energy acquisition from the environment, or cause individuals to allocate energy to adaptive or compensatory responses, ultimately altering their energy budget (Pirotta *et al.,* 2018a). In such cases, bioenergetics can also be used to predict the long-term consequences of exposure to anthropogenic stressors, particularly when measuring these effects empirically is unfeasible (e.g. for long-lived, wide-ranging species) (Costa *et al.,* 2016). As a result, bioenergetic modelling has become a prominent tool for both theoretical and applied ecophysiologists.

Here, a bioenergetic (or energy budget) model is defined as any mechanistic model where the principles of metabolic ecology are used to describe how an individual animal acquires energy from food resources (i.e. energy intake) and allocates assimilated energy to various life history functions (i.e. energy costs, including maintenance and survival, growth and reproduction) (Brown *et al.,* 2004; Johnston *et al.,* 2019; Kooijman, 2010; Sibly *et al.,* 2013). Energy intake can be either a model input, when allocation is modelled dynamically, or a model output, derived from energy requirements and the mechanisms involved in the feeding process. Acquisition and allocation can also be modelled to vary as a function of an individual’s state and the state of the environment (van der Meer, 2006).

Within the bioenergetic literature, there is considerable variation in how individual energy budgets are modelled (for an in-depth discussion of the interconnections and fundamental differences among approaches, see van der Meer, 2006 and Nisbet *et al*., 2012). ‘Traditional’ bioenergetic models (*sensu*  Nisbet *et al.,* 2012) describe energy acquisition from feeding and its partitioning among maintenance, activity, growth, reproduction and excretion; the advantage being that these processes have a clear empirical interpretation, which facilitates measuring them using explicit units, but the resulting models are often parameter-rich and hard to generalize across species. These models generally follow a hierarchical allocation, as proposed by Sibly *et al.* (2013) and extended by Beltran *et al.* (2017) and Gallagher *et al.* (2021a), whereby an individual expends assimilated energy in order of the importance of the processes to survival, that is, for maintenance, thermoregulation, locomotion, growth, reproduction and energy storage (up to an optimal amount of reserves). In contrast, Dynamic Energy Budget (DEB) theory (Kooijman, 2010) considers these same processes from a formal and more general perspective, using fundamental principles of mass–energy balance to relate sub-organismal (biochemical, genetic and physiological) processes to organismal performance (Martin *et al.,* 2012; Nisbet *et al.,* 2012; van der Meer, 2006). In turn, this generality results in abstract concepts that are harder to measure empirically. DEB models also follow a strict order and priority of energy allocation to different life functions (Kooijman, 2010). The energy dynamics of three compartments with fixed biochemical composition are considered: structure, reserves and (for adults) reproductive buffer. The energy intake via the food ingested is first deposited in the reserve buffer (Kooijman, 2010; Nisbet *et al.,* 2000). Throughout life, a constant fraction (kappa) is allocated to maintenance and somatic growth, while the remaining fraction (1 - kappa) is directed towards development and maturation (for juveniles) and reproduction (in adults)—the so-called ‘kappa-rule’ (Kooijman, 2010). In light of these energy fluxes, a system of differential equations is used to update the individual’s state variables, which may lead to events such as death (if maintenance costs cannot be covered) or reproduction.

Some bioenergetic models focus on assessing individual energy budgets and their variation as a function of external conditions. Others aim to investigate some population-level processes, e.g. a population’s overall prey consumption, the underlying demographic rates or the effects of changing extrinsic conditions (Johnston *et al.,* 2019; Sibly *et al.,* 2013). Individual energy budget calculations can be scaled to the population level in various ways. A distinction is introduced here between accounting bioenergetic models, which aim to predict the rate of energy intake from the environment, and dynamic bioenergetic models, which explicitly describe energy intake and allocation at a finer temporal scale. Accounting models combine individual energy budgets with estimates of population size to compute the overall energy requirements and prey consumption of a population at a particular moment in time (e.g. Acevedo and Urbán, 2021). In contrast, dynamic models allow for the investigation of the effects of variations in energy intake on different end points, e.g. average energy dynamics, vital rates and population dynamics (with or without density dependence). Simulations of population dynamics have then been achieved using various population modelling approaches (e.g. matrix models or physiologically structured population models; e.g. Klanjscek *et al.,* 2006; De Roos, 2008).

In recent years, bioenergetic models have increasingly been developed within the context of individual-based models (IBMs; also referred to as agent-based models) (Martin *et al.,* 2012; Mortensen *et al.,* 2021; Sibly *et al.,* 2013), which facilitate the inclusion of individual variation, local interactions and adaptation (Martin *et al.,* 2012). In IBMs, the responses of individuals to their internal state and external drivers (as represented, for example, by a mapped, ecological landscape) are simulated, resulting in emergent population dynamics (Grimm and Railsback, 2013). Because food is an important component of such modelled landscapes, and nutritional state responds faster to food availability than population density does, this integration is particularly useful for predicting the effects of changing conditions on populations (e.g. following anthropogenic disturbance or environmental change) (Sibly *et al.,* 2013). Moreover, the rate at which an individual acquires and uses energy can be modelled to vary with sex, age, activity state and experience (Sibly *et al.,* 2013). An important consideration when scaling to the population level (using any of the approaches mentioned) is whether the target population is at, or close to, carrying capacity, because density-dependent processes can affect the prey base and, in turn, individual health and vital rates (Hin *et al.,* 2021).

**Figure 1:**
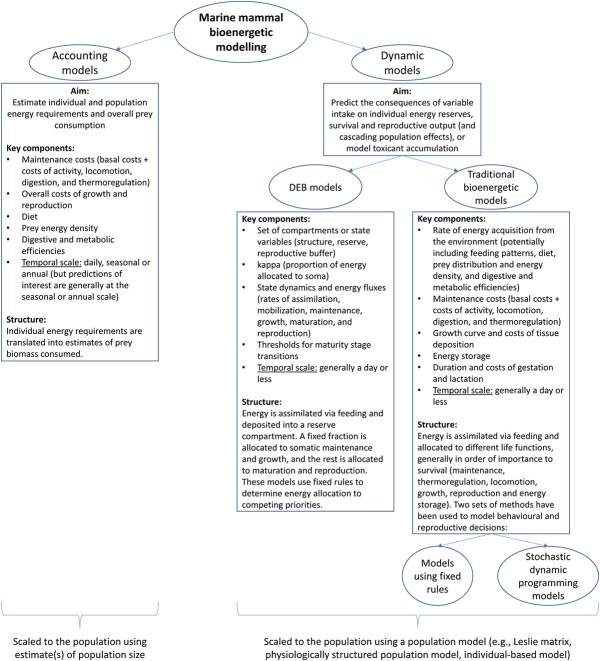
The variety of approaches used for marine mammal bioenergetic modelling.

Bioenergetic modelling has a long history of applications in marine mammal science, due to the variety of anthropogenic stressors that threaten the persistence of these species, and their life history characteristics, which make direct empirical measurements of individual- and population-level effects often unfeasible. In this review, I will first describe the early examples of accounting models for estimating prey consumption in marine mammal populations, highlighting the components that remain relevant in recent model developments. I will then move to dynamic models, which simulate the allocation of the energy assimilated from feeding to survival, growth and reproduction. I will distil the critical building blocks required to develop such models and describe how these have been informed for marine mammals (Fig. 1). In particular, recent developments in marine mammal science have been driven by the need to assess the population-level effects of stressors that have sublethal effects on individuals. The population consequences of disturbance (PCoD) conceptual framework describes how disturbance-induced changes in individual behaviour and physiology can affect population dynamics by compromising the health status of an individual, and thus its ability to survive and reproduce successfully (see review in Pirotta *et al.,* 2018b). While health encompasses multiple aspects of an individual’s physiological status, most PCoD implementations to date have focused on the changes in a female’s time-energy budget and the consequences on her ability to sustain the costs of maintenance (and thus survival) and reproduction (Keen *et al.,* 2021; Pirotta *et al.,* 2018b). Bioenergetic models represent an ideal tool to mechanistically capture this energetic pathway. It should be noted that, while there is a vast branch of marine mammal bioenergetics that has applied empirical and theoretical approaches to estimate the costs of different behaviours and life functions (e.g. Boyd *et al.,* 1993; Castellini *et al.,* 1992; Costa and Maresh, 2017; Fahlman *et al.,* 2016; Goldbogen *et al.,* 2011; Oftedal, 1997; Potvin *et al.,* 2012; Williams, 1999; Williams *et al.,* 2017), contributing critically to inform the parameters of bioenergetic models, these studies were outside the scope of the review.

## Bioenergetic accounting models of population energy requirements and resource consumption

The first examples of marine mammal bioenergetic models analysed the energy budgets of baleen whales (Lockyer, 1981a), sperm whales *Physeter macrocephalus* (Lockyer, 1981b), long-finned pilot whales *Globicephala melas* (Lockyer, 1993) and pinnipeds (Lavigne *et al.,* 1982) and aimed to shed light on their foraging ecology and energy requirements. These seminal works collated the knowledge available at the time, often derived from catch data, on diet, feeding rates, metabolism, growth, reproduction, seasonal dynamics and accumulation of reserves, and provided the theoretical and empirical basis for many subsequent modelling efforts.

Similar evaluations of populations’ overall energy requirements and prey consumption have continued over the years (Banas *et al.,* 2021; Bejarano *et al.,* 2017; Benoit-Bird, 2004; Costa *et al.,* 1989; Fortune *et al.,* 2013; Gallagher *et al.,* 2018; Guilpin *et al.,* 2019; Kriete, 1995; Lockyer, 2007; Malavear, 2002; McHuron *et al.,* 2017b; Noren, 2011; Noren *et al.,* 2012, 2014; Rechsteiner *et al.,* 2013; Reisinger *et al.,* 2011; Williams *et al.,* 2004; Winship *et al.,* 2002). Often, accounting models have been developed with the applied management goal of quantifying the levels of predation on prey stocks and potential competition with local fisheries (Acevedo and Urbán, 2021; Boyd, 2002; Cornick *et al.,* 2006; Faure *et al.,* 2021; Forcada *et al.,* 2009; Markussen *et al.,* 1992; McHuron *et al.,* 2020; Mohn and Bowen, 1996; Nilssen *et al.,* 2014; Olesiuk, 1993; Queiros *et al.,* 2018; Trzcinski *et al.,* 2006; Weise and Harvey, 2008).

**Figure 2:**
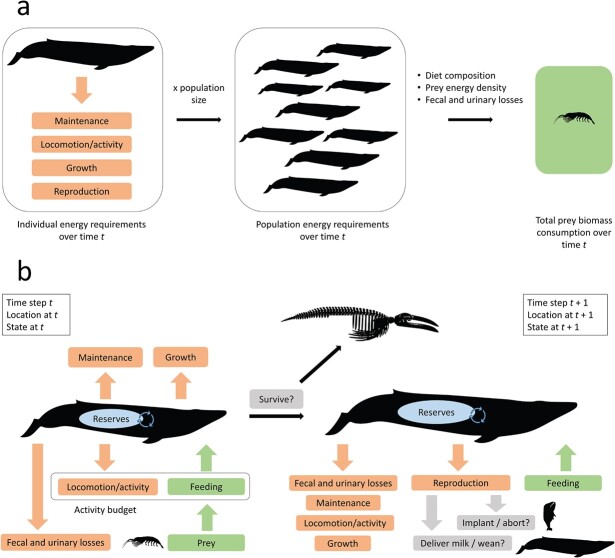
Schematic representations of (**a**) an accounting bioenergetic model and (**b**) a ‘traditional’ dynamic bioenergetic model (*sensu*  Nisbet *et al.,* 2012). In (a), individual energy requirements over some temporal interval of interest *t* (in orange) are scaled to the population using an estimate of population size, and then converted to total biomass of prey consumed by the population (in green) from information on diet, energy density and faecal and urinary energy losses. In (b), individuals are followed across time steps *t*, at which their location, energetic state and size are updated. Orange boxes indicate energetic costs and losses, green boxes represent the energy intake process and blue ellipses are used for an individual’s energy reserves. Here, maintenance includes thermoregulatory costs and the heat increment of feeding. At each time step, an individual’s vital rates (survival and reproduction) can also vary, as indicated in the grey boxes. For simplicity, potential reproductive decisions are only visualized for time step *t* + 1.

These studies used a variety of approaches to capture the costs incurred by individuals (Fig. 2a). Some examples calculated individual energy requirements using a combination of theoretical estimates of basal metabolic rate, derived from Kleiber (1975)’s allometric formula, and assumptions on how metabolic rate scales during activity (and in different activity states) (e.g. Boyd, 2002; Guilpin *et al.,* 2019; Noren, 2011; Winship *et al.,* 2002). In contrast, other studies used empirical measurements of metabolic rate obtained either in the field or in captivity (e.g. Costa *et al.,* 1989; Kriete, 1995; McHuron *et al.,* 2020). The term field metabolic rate (FMR) is generally used to indicate the metabolic rate that integrates the energy expended during all activities over the sampling period. When explicitly distinguishing among activity states, estimates of an individual’s activity budget have been used to partition energy expenditure over some unit of time (e.g. Costa *et al.,* 1989; Fortune *et al.,* 2013; Guilpin *et al.,* 2019). In line with the bioenergetic scheme proposed by Lavigne *et al.* (1982), some studies explicitly accounted for specific dynamic action (also referred to as heat increment of feeding), representing digestion costs (e.g. Fortune *et al.,* 2013; Winship *et al.,* 2002). In contrast, estimates of FMR from free-ranging animals are generally assumed to include digestion costs (e.g. McHuron *et al.,* 2020). The costs associated with production (i.e. structural growth, or tissue deposition more generally) and reproduction (gestation and lactation) have also been modelled separately in some cases (e.g. McHuron *et al.,* 2020); however, the latter have generally been considered in their totality (e.g. the overall energy required to bring a calf/pup to weaning, given birth and weaning sizes), as opposed to the corresponding day-by-day investments. In some examples, separate requirement estimates for individuals in different, age, sex or reproductive class were generated (e.g. McHuron *et al.,* 2020). Details of how energetic costs have been estimated for different marine mammal species are provided below, as part of the section ‘Key components of dynamic bioenergetic models’.

Individual requirements were then generally scaled to the population using estimates of population size (either single or multiple estimates, e.g. over multiple years), and converted to estimates of biomass consumed for different prey species using data on diet composition (e.g. from scat or stomach-content analysis) and prey energy density (Fig. 2a). The gross energy densities of many prey species have become increasingly available over the years (e.g. Lawson *et al.,* 1998; Spitz and Jouma’a, 2013), even though further studies are needed to evaluate variations in space and time and different approaches can limit comparability. Not all ingested energy is retained and available to fuel energetic requirements; digestible (or digestive) efficiency is defined as the proportion of gross ingested energy that is available after faecal loss, while metabolic efficiency also accounts for urinary losses (Lavigne *et al.,* 1982; Worthy, 2001). Both efficiencies are affected by the biochemical composition of the prey species (Schneider and Flatt, 1975). The term ‘assimilation efficiency’ has been used as a synonym of either digestive efficiency (e.g. in pinniped studies; the correct usage) or metabolic efficiency (e.g. in many cetacean studies).

It is important to note that, given their objective, these accounting models estimate energy requirements based on costs, which might not reflect actual energy intake in environments with variable resource availability. Moreover, accounting models do not discuss the differential allocation of energy to competing priorities (Fig. 1); for example, how a female should partition excess energy to her own body stores vs. providing it to her offspring in the form of milk. Such prioritization becomes critical when assessing the dynamic use of energy under changing external and internal conditions.

## Marine mammal dynamic bioenergetic modelling

Accounting models do not require input on the rate of energy intake from the environment, since their goal is to predict this value in light of individual energy requirements and to quantify a population’s potential prey consumption. In contrast, dynamic bioenergetic models explicitly describe energy intake and the allocation of acquired energy to different activities and life functions at a finer temporal scale (Fig. 1). Their primary objective is to understand how intake rate and allocation might change as external conditions vary, and to predict the consequences for an individual’s energy reserves and reproductive decisions (Costa *et al.,* 2016; McHuron *et al.,* 2017a; Sibly *et al.,* 2013). For example, climatic oscillations or anthropogenic climate change might lead to alterations in the abundance and availability of prey in the environment, which will affect energy intake and potentially the costs of foraging (e.g. Gallagher *et al.,* 2022). Human activities might also modify an individual’s energy budget by interfering with its behaviour, for example if they cause expensive avoidance responses or disturbance during feeding, reducing energy acquisition (e.g. McHuron *et al.,* 2021). These external drivers will interact with internal motivations (e.g. to grow or to reproduce) to influence an individual’s decision-making, its energy reserves and fitness and, ultimately, the dynamics of the population (Pirotta *et al.,* 2018b). In addition to assessing the effects of environmental change and anthropogenic disturbance, dynamic bioenergetic models have also been used to study the accumulation of persistent organic pollutants over the lifetime of individuals (e.g. Hickie *et al.,* 2000), since bioenergetic processes at different life history stages affect the uptake, metabolization and excretion of toxic compounds.

In marine mammal science, early dynamic models have been used to explore the allocation of energy reserves during periods of nutritional limitation (e.g. in the post-weaning phase for pinnipeds; Noren and Mangel, 2004; Noren *et al.,* 2009). These models have also found extensive use in recent years as tools to inform potential management and conservation strategies. They support predictions of the population-level effect of various stressors for these long-lived species (Costa *et al.,* 2016; Pirotta *et al.,* 2018b) where waiting for a change in population abundance to be detectable empirically is not compatible with conservation objectives (Taylor *et al.,* 2007). For example, Molnár and colleagues developed a DEB model for polar bears *Ursus maritimus* (Molnár *et al.,* 2010) and used it together with a model for body composition (Molnár *et al.,* 2009) to predict the effects of changes in duration of the fasting period due to climate change on litter size, cub recruitment and adult survival (Molnár *et al.,* 2011; Molnár *et al.,* 2020). Similarly, Udevitz *et al.* (2017) used the bioenergetic model by Noren *et al.* (2012, 2014) to assess how changes in sea ice availability could affect movements and activity patterns of Pacific walruses *Odobenus rosmarus*, while Beltran *et al.* (2017) investigated the consequences of a hypothetical reduction in food availability for Weddell seals *Leptonychotes weddellii*. Marine mammal bioenergetic modelling has also been used to study (theoretically) the bioaccumulation and vertical transfer of toxicants and the transmission of infectious diseases (Hickie *et al.,* 2000; Hickie *et al.,* 2005; Hickie *et al.,* 2007; Hickie *et al.,* 2013; Klanjscek *et al.,* 2007; Noonburg *et al.,* 2010; Silva *et al.,* 2020). Other applications include the effects of fisheries on the prey base (Wiedenmann *et al.,* 2011) and the cost of predation risk (Srinivasan *et al.,* 2018).

The most widespread use of dynamic bioenergetic models in the past decade has been in the context (either implicit or explicit) of assessing the long-term consequences of sublethal anthropogenic disturbance on individuals or populations under the PCoD framework, which has included applications to pinnipeds (Goedegebuure *et al.,* 2018; McHuron *et al.,* 2017a, 2018), harbour porpoises *Phocoena phocoena* (Gallagher *et al.,* 2021a; Harwood *et al.,* 2020; Nabe-Nielsen *et al.,* 2014; Nabe-Nielsen *et al.,* 2018), delphinids (Hin *et al.,* 2019; New *et al.,* 2013b; Pirotta *et al.,* 2014; Pirotta *et al.,* 2015, 2020; Reed *et al.,* 2020; Williams *et al.,* 2006), beaked whales (New *et al.,* 2013a), baleen whales (Braithwaite *et al.,* 2015; Christiansen and Lusseau, 2015; Dunlop *et al.,* 2021; Guilpin *et al.,* 2020; McHuron *et al.,* 2021; Pirotta *et al.,* 2018a, 2019, 2021; Riekkola *et al.,* 2020; van der Hoop *et al.,* 2017; Villegas-Amtmann *et al.,* 2015; Villegas-Amtmann *et al.,* 2017), small- to medium-sized odontocetes (Noren *et al.,* 2017) and sperm whales (Farmer *et al.,* 2018b, 2018a).

## Key components of dynamic bioenergetic models

Dynamic bioenergetic models require information on the rate of energy acquisition from the environment and the accumulation of energy reserves, an estimate of maintenance costs (e.g. from FMR, which has been generally assumed not to include any growth and reproductive costs), a growth curve to predict mass and reserve accumulation abilities at different ages and the duration and costs of gestation and lactation (Pirotta *et al.,* 2018b) (Fig. 2b). This information may be available for the population or species of interest, but studies have borrowed data from related species, or species with a comparable life history, to fill knowledge gaps (Sibly *et al.,* 2013). The application of these models to the assessment of the consequences of stressors on individuals and populations and the typical scale of marine mammal responses to disturbance have also led to the use of finer temporal resolutions than accounting models (e.g. a day).

Some early PCoD models used an arbitrarily scaled energy currency, representing an individual’s underlying motivational state (Nabe-Nielsen *et al.,* 2014; New *et al.,* 2013b; Pirotta *et al.,* 2014; Pirotta *et al.,* 2015). While this approach requires less input information, in practice there have been substantial advantages in modelling energy budgets using an explicit unit (e.g. joules). For example, this provides a clearer interpretation of an individual’s predicted energetic state (which can be compared to empirical measurements), as well as a more straightforward conversion of prey intake to energy, and of energy to reproduction (Pirotta *et al.,* 2018b).

Once individual energy budgets are in place, marine mammal bioenergetic modelling has used different methods to scale from individuals to populations. Some studies have only derived the distribution of vital rates (survival and reproductive success) or of toxicant concentrations across individuals, e.g. using IBMs (Christiansen and Lusseau, 2015; Hickie *et al.,* 2000, 2013; Hickie *et al.,* 2005; Hickie *et al.,* 2007; Molnár *et al.,* 2011; Molnár *et al.,* 2020; New *et al.,* 2013a; Pirotta *et al.,* 2019, 2020; Villegas-Amtmann *et al.,* 2015). Other studies have extended the analysis to the emerging dynamics of the population, e.g. using matrix models (Farmer *et al.,* 2018b), physiologically structured population models (Hin *et al.,* 2019) and, widely, IBMs (e.g. Gallagher *et al.,* 2021a; Goedegebuure *et al.,* 2018; McHuron *et al.,* 2018; Nabe-Nielsen *et al.,* 2018; Silva *et al.,* 2020; Villegas-Amtmann *et al.,* 2017). Very few examples have included the effects of density-dependent processes on modelled populations, either explicitly or as an emergent property of the interaction between predator consumption and prey availability (Gallagher *et al.,* 2021a; Goedegebuure *et al.,* 2018; Hin *et al.,* 2021; Nabe-Nielsen *et al.,* 2014; Nabe-Nielsen *et al.,* 2018; Silva *et al.,* 2020).

The basic building blocks of dynamic models (energy intake, maintenance costs, growth, reproduction and energy storage) have been informed using approaches with varying level of specificity and complexity, which are discussed in the next sections.

### Energy acquisition: feeding and prey

Energy acquisition through feeding has been modelled, in the simplest scenario, assuming a fixed energy intake rate that does not explicitly consider prey availability, abundance or energy density but is derived from estimates of energetic costs (e.g. Farmer *et al.,* 2018a). This intake rate has then been simulated to vary as a function of changing environmental conditions or disturbed feeding. For example, Farmer *et al.* (2018a) modelled the effects of disturbance as a caloric deficit to be covered using energy reserves. Alternatively, other studies have used a functional response relationship, describing intake rate given the availability of prey in the environment. Functional responses can range from purely phenomenological to mechanistic (Jeschke *et al.,* 2002; Smout and Lindstrøm, 2007), even though suitable data to estimate empirical relationships are often lacking for marine mammals. For example, Hin *et al.* (2019) assumed a linear functional relationship between prey availability and the maximum rate of energy intake; they then scaled ingestion rate as a function of structural mass, body condition and age. Species- or context-specific feeding strategies have also been simulated (e.g. Guilpin *et al.,* 2019; McHuron *et al.,* 2018; Pirotta *et al.,* 2019). In these cases, functional response relationships emerge indirectly from the mechanisms and constraints involved in prey acquisition. For example, Pirotta *et al.* (2018a, 2019) explicitly modelled individual blue whales *Balaenoptera musculus* encountering prey patches as they moved in the environment, and, within patches, their lunging behaviour, incorporating data on diving, lunge rates, engulfment volumes, prey densities, digestion capacity and diel variation. Theoretical foraging models have been developed in other cases: for example, Nabe-Nielsen *et al.* (2014) and Nabe-Nielsen *et al.* (2018) used the model described in Nabe-Nielsen *et al.* (2013) to simulate harbour porpoise exploratory movements and spatial memory of previous food patches; food consumption in a patch was then linearly related to the resource level at that patch, scaled by porpoise body condition. In general, more mechanistic approaches have supported an easier identification of knowledge gaps, which can inform targeted data collection, and have facilitated the simulation of changing conditions that may affect specific aspects of the feeding process. However, a coherent discussion of how to capture foraging behaviour in bioenergetic models is largely lacking, particularly with regard to the competing constraints operating on feeding: for example, the amount of time dedicated to feeding might be limited by prey processing (e.g. digestion) or diving capacity, while energetic state can also have feedback effects on feeding abilities (e.g. via changes in buoyancy and thermoregulatory costs) (Rosen *et al.,* 2007).

Quantifying the spatio-temporal variability in the abundance, availability, energy density and digestibility of prey resources is challenging for marine mammals. Some bioenergetic models have used marine mammal distribution or behaviour (e.g. feeding rates) as a proxy of the underlying resources (e.g. McHuron *et al.,* 2018; Nabe-Nielsen *et al.,* 2014; Nabe-Nielsen *et al.,* 2018), while others have acknowledged the data gap and explored the effects of different resource levels on model predictions (e.g. Goedegebuure *et al.,* 2018; Hin *et al.,* 2019). A more explicit representation of the environment has been achieved in cases where prey species are targeted by systematic data collection, or where proxies of environmental productivity can be used in conjunction with targeted sampling of prey patches (e.g. Guilpin *et al.,* 2019, 2020; Pirotta *et al.,* 2019, 2021). However, accurate prey modelling is more challenging for species with a generalist diet or targeting prey items with no commercial value. Sensitivity analyses have demonstrated that this component has a disproportionately large influence on any model outcome (e.g. Pirotta *et al.,* 2018a), because the environment affects baseline behavioural and reproductive strategies (Pirotta *et al.,* 2020), as well as resilience and compensatory abilities (Hin *et al.,* 2019; Pirotta *et al.,* 2019).

### Energy costs: maintenance, structural growth and reproduction

In terms of energy expended for maintenance (here intended to include basal costs and the costs of locomotion, thermoregulation, and digestion), dynamic bioenergetic models have used similar strategies to the ones described above for accounting models, relying on a combination of theoretical assumptions of how metabolic rates may vary under activity and empirical measurements of metabolic rates in captivity or in the wild. Similarly, the costs of different behavioural states have been either grouped and summarized using FMR (e.g. McHuron *et al.,* 2018), or treated separately (e.g. Pirotta *et al.,* 2019). Explicitly modelling different behaviours has proven useful in cases where an individual’s sensitivity to stressors is state-dependent (e.g. Pirotta *et al.,* 2021), where exposure to a disturbance source causes changes in the activity budget (e.g. Pirotta *et al.,* 2014), or if the species’ life history involves seasonal patterns characterized by highly variable activity states (e.g. the migration between feeding and breeding grounds; Pirotta *et al.,* 2018b). Moreover, because marine mammals occur in patchy environments, are driven by spatially explicit processes (e.g. the requirement to feed or reproduce in specific areas) and interact with heterogeneously distributed human activities, there are advantages in modelling movement explicitly. However, this additional complexity has required further data on activity budgets (including movement patterns) and activity-specific metabolic rates (e.g. Pirotta *et al.,* 2019). Alternatively, some studies have modelled the costs of locomotion using a theoretical approach based on drag and propulsion forces, i.e. the mechanical power required to move (Beltran *et al.,* 2017; Gallagher *et al.,* 2021a), while others have chosen a spatially implicit approach (Hin *et al.,* 2019). The costs associated with digestion have also been ignored in most models, or assumed to be part of FMR (but see Beltran *et al.,* 2017). Similarly, while most studies have assumed fixed thermoregulatory costs as part of FMR or assumed that animals operated in thermoneutral conditions, some have used theoretical calculations of heat transfer to explicitly model thermoregulation (Beltran *et al.,* 2017; Gallagher *et al.,* 2021a). A recent study that used a bioenergetic model to estimate heat losses in grey whale *Eschrichtius robustus* calves in comparison with FMR (derived from breathing rates) concluded that calves in good body condition do not require additional thermogenesis (Sumich, 2021). However, thermoregulatory costs and their variation with body condition, activity and the environment remain relevant for smaller species with larger surface area to volume ratios (Rosen *et al.,* 2007).

The temporal resolution of dynamic models has implications for how the investment of energy towards structural growth and reproduction is modelled, because, as noted above, most early studies reported total costs. In most marine mammal bioenergetic models to date, all costs associated with growth and reproduction have been treated separately from FMR. The energetic requirements for structural body growth per unit time (e.g. daily) have been generally calculated from estimates of growth rates (derived from a growth curve, e.g. a Von Bertalanffy length–age relationship fitted to data of a given species, paired with a length–mass relationship, and average body composition or estimated body condition), combined with the energy density of lean mass or the costs associated with tissue deposition (Hin *et al.,* 2019; McHuron *et al.,* 2018; Pirotta *et al.,* 2019). With the exception of studies strictly adhering to DEB theory (e.g. Silva *et al.,* 2020), where growth is an emergent property of the size of the reserve buffer, almost all marine mammal models have assumed that the rate of growth in structural mass follows the assumed growth curve exactly (see discussion below).

The total costs of gestation include the costs of depositing foetal tissue, which are usually derived from body size and chemical composition at birth (estimated either from a full-term foetus or a neonate), as well as the heat of gestation (i.e. all additional metabolic overheads associated with pregnancy, e.g. the costs of maintaining the placenta), which has been estimated mostly using Brody (1968)’s equation (Lockyer, 1993, 2007). Some studies have also modelled the energy contained in placental and uterine tissues (e.g. Hin *et al.,* 2019; McHuron *et al.,* 2018; Villegas-Amtmann *et al.,* 2015). Partitioning gestation costs at fine time scales (e.g. a day) over the course of pregnancy is challenging. In some cases, this has been based on the change in foetus size through pregnancy, resulting in smaller costs during early gestation (e.g. Hin *et al.,* 2019; Pirotta *et al.,* 2019; Silva *et al.,* 2020). In the absence of relevant data, these costs have been crudely partitioned by the time unit of the models or by coarse phases of pregnancy. Moreover, empirical data suggest that tissue deposition (i.e. foetus growth) can be regulated based on female condition (Christiansen *et al.,* 2014), but to date only one study has modelled foetal size dynamically (McHuron *et al.,* 2021). Some studies have also explicitly considered the additional drag costs imposed by pregnancy during locomotion (Pirotta *et al.,* 2019). Finally, while existing models have simply added the energy required to support gestation to other costs, empirical evidence suggests that several mechanisms may exist during pregnancy that allow individuals to compensate for the heat of gestation (e.g. metabolic depression; Sparling *et al.,* 2006).

The costs of lactation have been modelled following either a bottom-up or a top-down approach. Bottom-up approaches have calculated the amount of energy transferred from mother to offspring per unit of time from estimates of the offspring’s needs, i.e. the total amount of energy required for a calf or pup to reach weaning. This has been derived from birth and weaning masses, estimates of body composition and estimates of metabolic costs (e.g. calf or pup FMR), and partitioned based on the estimated duration of the lactation period (often approximate for cetacean species) (e.g. Villegas-Amtmann *et al.,* 2015). In some studies, the delivery of milk has been modelled explicitly (e.g. Hin *et al.,* 2019; Pirotta *et al.,* 2019; Silva *et al.,* 2020). These approaches also require an estimate of the efficiency of lactation in promoting mass gain. In contrast, top-down approaches have used estimates of the amount of milk a female produces (e.g. Beltran *et al.,* 2017). While measurements of milk output and intake volumes, energy density and chemical composition, and the associated changes in pup mass, are possible in pinnipeds, to some extent, information for cetaceans is more limited and often derived from catch data (Oftedal, 1997). Alternatively, milk quantities have been derived from the mass of the mammary tissue and the amount of milk produced per unit mass (Oftedal, 1997). Across approaches, the delivery of energy is generally assumed to follow some allocation rule based on female and offspring age, size and/or condition, which ultimately determine a female’s provisioning strategy (Gallagher *et al.,* 2021a; Hin *et al.,* 2019; McHuron *et al.,* 2018; New *et al.,* 2013a; Pirotta *et al.,* 2019).

### Energy storage

Reserve tissues are normally treated separately from structural growth, as energy stores are dynamically deposited or mobilized depending on the net intake in each time unit (note that, under the DEB framework, energy is assumed to be first temporarily stored in a reserve buffer and then mobilized towards various functions; Kooijman, 2010). In the majority of bioenergetic models to date, energy has been assumed to get stored in subcutaneous blubber or, more generally, in adipose tissues across the body, with few exceptions where the amount of carbohydrates, lipids and proteins in the body has been modelled explicitly (Farmer *et al.,* 2018a). As a result, relative body condition (e.g. the proportion of reserve mass to total body mass) or the absolute amount of blubber reserves have been generally used as the relevant state variable in bioenergetic models. However, some energy can also be derived from the catabolism of lean tissues (Bennett *et al.,* 2007; Rosen *et al.,* 2007), particularly when under thermoregulatory constraints (Worthy and Lavigne, 1987). Such processes may be particularly important for some deep-diving cetaceans (e.g. beaked and sperm whales), whose blubber is mainly composed of wax esters, which are difficult to metabolize and thus not ideal as energy storage (Koopman, 2007). The processes involved in storing and using stored energy have also been modelled with a varying degree of complexity; for example, some models have explicitly considered changes in blubber volume, requiring, among others, estimates of blubber lipid content, deposition and mobilization efficiency and energy density (Gallagher *et al.,* 2021a), while others have simply used estimates of blubber energy density to convert mass to energy and vice versa (McHuron *et al.,* 2018; Pirotta *et al.,* 2019). More generally, the dynamics of energy stores and associated signalling pathways in marine mammals are an area of active research (Derous *et al.,* 2021).

### Decision-making and energy-allocation strategies

Because the goal of bioenergetic modelling exercises is often to predict the effects of suboptimal energy intake (due to either reduced prey resources or disturbed feeding), models must simulate how individuals adjust their behaviour and reproduction in response to changing conditions, and, more generally, how they prioritize the allocation of energy to competing functions.

Animal strategies around behaviour, growth and reproduction vary depending on the interplay between the external environment and the internal state of an individual (including, for example, its energy reserves or age). Explicitly modelling the variation in individual decisions requires an understanding of these trade-offs from a fitness perspective (Houston and McNamara, 1999; Mangel and Clark, 1988). Dynamic state variable models, implemented via stochastic dynamic programming, have been used to explore the multidimensional matrix of optimal behavioural and reproductive decisions at any moment in the life of an individual, given the combination of internal and external state variables (McHuron *et al.,* 2017a, 2018, 2021; Pirotta *et al.,* 2018a, 2019, 2022). As an alternative, fixed rules (e.g. based on thresholds of state variables) have been developed (Hin *et al.,* 2019) (Fig. 1). Pirotta *et al.* (2020) provide a comparison of the two approaches to model reproductive decisions, using the same model with alternative formulations, and discuss their appropriateness in different environmental contexts. A comparable discussion around structural growth is lacking. Most models have assumed that structural growth is a fixed cost (but see: Wiedenmann *et al.,* 2011; Gallagher *et al.,* 2021a) and thus included it as part of the baseline costs incurred by an individual, but suboptimal resources may result in reduced or delayed growth (Stewart *et al.,* 2021; Trites and Donnelly, 2003), which might affect the age at which individuals mature as well as metabolic rates (Nylin and Gotthard, 1998; Stearns, 1992). This assumption has strong implications on model predictions, because structural size affects the amount of energy reserves an individual can accumulate and thus its resilience to periods of reduced feeding and ability to reproduce successfully.

Fundamentally, these processes relate to the principles that are assumed to govern energy allocation towards different life functions. Some marine mammal bioenergetic models have adhered to DEB theory and used the kappa-rule (Goedegebuure *et al.,* 2018; Silva *et al.,* 2020) (Fig. 1). Others have preferred approaches comparable to the hierarchical allocation rules described in Sibly *et al.* (2013). Allocation priorities are unknown and may vary between species, individuals and over time; therefore, as noted by Sibly *et al.* (2013), ‘[f]urther work by evolutionary biologists is needed’. Meanwhile, phenomenological patterns of individual growth and reproductive investment (e.g. derived from photogrammetry data; Christiansen *et al.,* 2018) can be used to guide modelling efforts.

## Discussion and conclusions

A variety of approaches have been followed to develop bioenergetic models for marine mammals over the past four decades. Such variety reflects the large range of life histories exhibited by these species, determining the components of their metabolic ecology that require explicit inclusion, and the diversity of management and conservation applications and needs, imposing different model complexity, granularity and structure. Moreover, different simplifying assumptions have been used in different models to ensure they remained tractable. This flexibility in modelling strategy has allowed models to be tailored to specific questions, but at the cost of generality. While broad strands of comparable approaches can be identified, most models have been developed *ad hoc* for a specific case study, and, as such, cannot be easily extended to other species (or even populations of the same species) in other contexts. In this sense, DEB theory has the advantage of formalizing energy budgets in terms of first principles (Kooijman, 2010), while retaining some of the desirable flexibility when embedded into IBMs (Martin *et al.,* 2012). However, there is debate surrounding the most appropriate way to represent energy allocation and prioritization (Sibly *et al.,* 2013), and the parameters of DEB models are harder to inform directly using empirical studies (Nisbet *et al.,* 2012). Moreover, including additional processes that are not covered in the original DEB model structure (e.g. the costs of locomotion) is not straightforward.

Whichever approach is taken, it is critical that extensive and rigorous documentation is provided to support modelling decisions and the choice of parameter values. Schematic representations of model structure and bioenergetic processes, mathematical equations, pseudocode (i.e. the plain language description of the steps in the model), tables of parameter values and associated units, uncertainty and references and the visualization of inbuilt relationships (e.g. functional responses, fitness functions, and growth curves) have all been used to concisely document bioenergetic models (e.g. Beltran *et al.,* 2017; Gallagher *et al.,* 2021a; Hin *et al.,* 2019; McHuron *et al.,* 2020; Pirotta *et al.,* 2018a). When bioenergetic models are formulated as IBMs, a formal protocol exists on how to compile and structure this documentation (the Overview, Design concepts and Details, or ODD, protocol; Grimm *et al.,* 2006, 2020), which has been used to describe some marine mammal bioenergetic models (e.g. Gallagher *et al.,* 2021a). The ODD protocol can also be applied to other modelling approaches (e.g. Meli *et al.,* 2014). Given the large number of parameters that are normally required for the development of these models, information has often been borrowed from the literature; however, not all studies report the primary references or explicitly highlight if these used guestimates or expert opinions, rather than empirically derived values. DEB theory provides tools for the estimation of standard model parameters, based on empirical data and theoretical assumptions (Martin *et al.,* 2012). Parameter values are also available from the Add-my-Pet database (https://www.bio.vu.nl/thb/deb/deblab/add_my_pet/), which allows comparisons across species and taxa.

Validating bioenergetic models remains challenging, and a thorough discussion of the issue is beyond the scope of this review. Most existing bioenergetic models are not fitted directly to data, but there has been recent progress in fitting complex mechanistic models using approximate Bayesian computation or emulation (Hooten *et al.,* 2020). Pattern-oriented modelling can be used to guide model design, calibration and selection so that multiple emergent properties of a model reproduce patterns observed in the real system (Gallagher *et al.,* 2021b; Grimm and Railsback, 2012). Uncertainty at all stages of the modelling process (structuring, parameterization, input values, system stochasticity) should also be quantified and appropriately propagated (Harwood and Stokes, 2003; Milner-Gulland and Shea, 2017). In particular, sensitivity analyses have been a useful tool to help ‘determine the robustness of conclusions to plausible violation of model assumptions and variation in the inputs’ and highlight the most critical research gaps to be filled (Pirotta *et al.,* 2018b).

From a conceptual perspective, many gaps remain in our knowledge. Aside from the issue of how energy is allocated to competing life functions and what state variables should be used to best represent energy reserves, there is large uncertainty on how metabolic rate scales with activity and environmental conditions, the functional responses and feeding constraints, the patterns and flexibility of structural growth, the regulation of reproduction as a function of nutritional status, the dynamics of energy reserves and associated signalling pathways, the variation in maintenance costs (e.g. due to variable thermoregulation and digestion) or the effects of physiological status (e.g. stress levels and immune function) on metabolic ecology. Importantly, while existing models have disproportionately focused on the cost side of the energy budget equation, marine mammal bioenergetic research will be severely hampered until a better understanding of the dynamics and distribution of prey resources (i.e. their availability, accessibility, abundance, energy content and digestibility) is achieved. Tackling this critical source of uncertainty will involve a discussion across multiple disciplines. Another aspect that requires further scrutiny with the help of the physiological community is the choice of an appropriate temporal scale for bioenergetic models and how the overall costs of certain life functions (e.g. lactation) are partitioned as a result. Sensitivity analyses can help in the exploration of some of these uncertainties. While only a limited subset of existing bioenergetic studies included a formal sensitivity analysis, results have been consistent: parameters associated with the prey (e.g. its availability, density and energy content), the feeding and digestion process (e.g. feeding rates and assimilation efficiency), energy expenditure in different activity states (e.g. FMR), morphology (e.g. individual size and energy storage abilities) and the lactation process (e.g. the amount of milk delivered as a function of female condition and the duration of lactation) have been invariably found to strongly affect predicted outcomes (Bejarano *et al.,* 2017; Beltran *et al.,* 2017; Gallagher *et al.,* 2021a; Goedegebuure *et al.,* 2018; Guilpin *et al.,* 2019, 2020; Harwood *et al.,* 2020; McHuron *et al.,* 2017a, 2020; Molnár *et al.,* 2009; Nabe-Nielsen *et al.,* 2018; Pirotta *et al.,* 2018a). One study has also found the relationship between available energy reserves and survival probability to be particularly influential on model predictions (Gallagher *et al.,* 2021a). These results provide a roadmap to guide the prioritization of future research efforts in order to reduce uncertainties most effectively. The selection of target species and regions for bioenergetic modelling efforts to date has been largely driven by the availability of funding in different jurisdictions and by management needs. A critical evaluation of the populations and associated stressors that would most benefit from a bioenergetic modelling approach will also be important for prioritizing future research.

In conclusion, 40 years after the development of the first equations to describe cetacean and pinniped energy intake and costs, the field of marine mammal bioenergetic modelling is arguably at its golden age. Species- or population-specific models are increasingly available, with important applications to predict the consequences of environmental change and human impacts on population dynamics. However, the wide range of potential analytical structures to choose from and the many outstanding empirical uncertainties still present a challenge for bioenergetic modellers. The development of best practices for modelling and data collection (see the other papers in this Special Issue) will support coordinated and coherent efforts, ultimately providing powerful tools to inform effective management and conservation strategies.

## Funding

This work was supported by the U.S. Office of Naval Research [grant number N000142012392] and the Marine Mammal Commission [award number MMC21-056].

## Data availability

Not applicable.
